# Evaluation of JQ1 Combined With Docetaxel for the Treatment of Prostate Cancer Cells in 2D- and 3D-Culture Systems

**DOI:** 10.3389/fphar.2022.839620

**Published:** 2022-02-03

**Authors:** Yipeng Xu, Gabriela Pachnikova, Dorothea Przybilla, Reinhold Schäfer, Yingying Cui, Dan Zhou, Zihao Chen, An Zhao, Ulrich Keilholz

**Affiliations:** ^1^ Department of Urology, The Cancer Hospital of the University of Chinese Academy of Sciences (Zhejiang Cancer Hospital), Hangzhou, China; ^2^ The Key Laboratory of Zhejiang Province for Aptamers and Theranostics, Hangzhou, China; ^3^ Institute of Basic Medicine and Cancer (IBMC), Chinese Academy of Sciences, Hangzhou, China; ^4^ Comprehensive Cancer Center, Charité—Universitätsmedizin Berlin, Corporate Member of Freie Universität Berlin, Humboldt-Universität zu Berlin and Berlin Institute of Health, Berlin, Germany; ^5^ Department of Urology, Southern Medical University, Guangzhou, China; ^6^ Experimental Research Center, Cancer Hospital of the University of Chinese Academy of Sciences (Zhejiang Cancer Hospital), Hangzhou, China; ^7^ German Cancer Consortium (DKTK), Heidelberg, Germany

**Keywords:** Prostate Cancer, JQ1, Docetaxel, Combination Treatment, 3D Culture Spheroid

## Abstract

**Introduction:** Prostate cancer (PCa) is dependent on coupled androgen-androgen receptor (AR) signaling for growth and progression. Significant efforts have been made in this research field, as hormonal therapies have greatly improved the survival of patients with metastatic PCa (mPCa). The drug treatment agent JQ1, which potently abrogates bromodomain 4 (BRD4) localization to the AR target loci and therefore significantly impairs AR-mediated gene transcription, is a potent therapeutic option for patients with advanced PCa. In this study, we aimed to investigate the inhibitory effect of JQ1 combined with docetaxel on PCa cells *in vitro* for the first time. Furthermore, the 3D spheroid culture system was modeled to more accurately simulate the response of PCa cells to drugs.

**Methods:** We established and measured 3D LNCaP spheroids *in vitro* in order to evaluate the susceptibility of 2D- and 3D-cultured LNCaP cells exposed to the same anti-cancer drug.

**Results:** We demonstrated that JQ1 was an effective drug for promoting cell inhibition after docetaxel treatment in 2D- and 3D- cultured LNCaP cells. Inhibition of 3D cultured formation in the combined treatment group was significantly higher than that in docetaxel or JQ1 alone. Under the same conditions of drug solubility, the drug resistance of 3D spheroids was significantly higher than that of 2D cells. Moreover, d_max_ and lg volume were suitable parameters for LNCaP cells/spheroid size displaying and evaluating cell viability.

**Conclusion:** 3D cultured spheroids of PCa are an effective tool for studying PCa drug trials. JQ1 combined with docetaxel may be an effective treatment for advanced PCa. This combination therapy strategy deserves further evaluation in clinical trials.

## Introduction

Prostate cancer (PCa) remains the most prevalent cancer in men globally, with approximately 1.6 million newly diagnosed cases and 366,000 deaths each year (Pernar et al., 2018). PCa was once considered a common malignancy in elderly males, while recent studies have shown that the incidence of PCa in young males is significantly increasing (Bleyer et al., 2020). This indicates that the global burden of PCa may become more substantial in the future.

PCa is unique in its dependence on the androgen receptor (AR) signaling pathway for growth and progression (Lim et al., 2020). As such, androgen deprivation therapy (ADT) has been considered the backbone of treatment for advanced and metastatic PCa (Nader et al., 2018). During the initial treatment course, most patients with PCa respond well to medical castration. However, almost all non-early stage PCa patients become castration-resistant over time and develop advanced PCa of castration-resistant PCa (CRPC) or metastatic CRPC (mCRPC), wherein PCa cells develop mechanisms to proliferate despite castrate levels of testosterone (Lim et al., 2020). Although two next-generation hormonal drugs abiraterone (Stein et al., 2012) and enzalutamide (Scher et al., 2012) have been found to significantly improve the outcome of mCRPC, the durable responses were limited (Khalaf et al., 2019).

In contrast, docetaxel is the first chemotherapy agent to extend the overall survival of patients with mCRPC (Tannock et al., 2004). Several studies have also reported significant synergistic effects of docetaxel combined with estradiol or other drugs on advanced PCa (Figg et al., 2007; Tombal, 2007; Dahmani et al., 2010; Kuramoto et al., 2013; Jain et al., 2015). Recently, a new small molecule that functions downstream of AR, JQ1, has provided a new strategy for advanced PCa treatment (Zhang et al., 2017). Compared with hormonal drugs, JQ1 more potently abrogates bromodomain and extra-terminal (BET) localization to AR target loci and inhibits AR-mediated gene transcription (Asangani et al., 2014). Moreover, the effects of JQ1 have been shown to be synergistically amplified by the addition of docetaxel *in vitro* and *in vivo* in esophageal adenocarcinoma (Song et al., 2020). These results suggest that JQ1 combined with docetaxel may improve the therapeutic effect for advanced PCa. Therefore, in this study, we aimed to investigate the inhibitory effect of JQ1 combined with docetaxel on PCa cells *in vitro* for the first time, and a 3D spheroid culture system was modeled to more accurately simulate the response of PCa cells to drugs.

## Methods

### Cells Culture and Reagents

The LNCaP cell line was kindly gifted from the Urology Department of Charité Campus Mitte. All the cells were cultured under sterile conditions in a humidified incubator with 5% CO_2_ at 37°C (Thermo Scientific; Massachusetts, United States), and were maintained in RPMI 1640 medium (Gibco; Texas, United States) supplemented with 10% fetal bovine serum (Gibco; Texas, United States) and 1% penicillin and streptomycin (stock 10,000 μg/ml each) (Life Technologies; New York, United States) according to the complete growth medium described on the ATCC website.

### 3D Embedded Cells/Spheroids Culture

The 3D cultured LNCaP cells/spheroids were initiated from 2D cultured LNCaP cells. From the third passage after cell thawing, a certain number of 2D cultured LNCaP cells were resuspended in Matrigel Matrix (Corning, New York, United States) and plated into the center of each well. Growth medium was exchanged every 3 days for cells seeded in 24 well plates (Corning; New York, United States), and half exchange of medium was applied to cells seeded in 96-well plates (Corning, New York, United States) every second day.

### Drug Preparation and Treatment

The agents were freshly prepared before the treatment experiments in the growth medium. The medium with a single agent was prepared as follows: 1) docetaxel (AbCam; Cambridge, United Kingdom) at final concentrations of 0.25 nM, 0.5, 1, 2, 4 nM, 8 and 16 nM; 2) JQ1 (Cayman Chemical, United States) at final concentrations of 8, 16, 32, 64, 128, and 256 nM. The medium of the mixture was prepared as follows: 1) 1 nM docetaxel combined with 128 nM JQ1; 2) 2 nM docetaxel combined with 128 nM JQ1). Culture medium without any agent was used as the negative control. The prepared medium was gently added to the cells (1 ml/well for 24 well plate, 200 ul/well for 96-well plate), and was changed every 3 days. The growth of cells or spheroids was observed using a TCS SPE confocal system microscope (Leica, Germany).

### Drug Interaction Testing by Checkerboard Assay

Checkerboard assays were used to determine the pairwise interactions between JQ1 and docetaxel. Serial 2-fold dilutions starting at 64/32 times the lowest drug concentration of the test agent were prepared and plated in 96-well clear plates (Corning, New York, United States) in the horizontal and vertical directions, respectively. Checkerboard results were assessed visually, with 100% inhibition as the endpoint. The initial determination of interactions between JQ1 and docetaxel used an abbreviated diagonal-sampling checkerboard method (Cokol-Cakmak and Cokol, 2019).

### Cell Viability Analysis

The CellTiter Glo assay (Promega, Wisconsin, United States) was performed to examine cell viability 5 days after the beginning of the treatment according to the manufacturer’s instructions using a VICTOR Nivo Multimode Microplate Reader.

### Characterization of Spheroids

Spheroids were imaged twice a week and later analyzed for their volume, log10 volume (lg volume), and long diameter (d_max_). According to the literature (Berrouet et al., 2020a), the LNCaP spheroid volume was calculated as V = π × d_max_ × d_min_^2^/6.

### Statistics

The normality tests of LNCaP cells/spheroid size were analyzed using GraphPad Prism 8 (GraphPad Software; CA, United States) software, including frequency distribution and Gaussian distribution (D’Agostino-Pearson omnibus normality test). The line charts and violin plots of spheroid parameters (d_max_, lg volume) were plotted using GraphPad Prism 8 software, as well as the unpaired t-tests of the spheroid formation parameters.

## Results

### Establishment and Measurement of 3D LNCaP Spheroids

**FIGURE 1:**
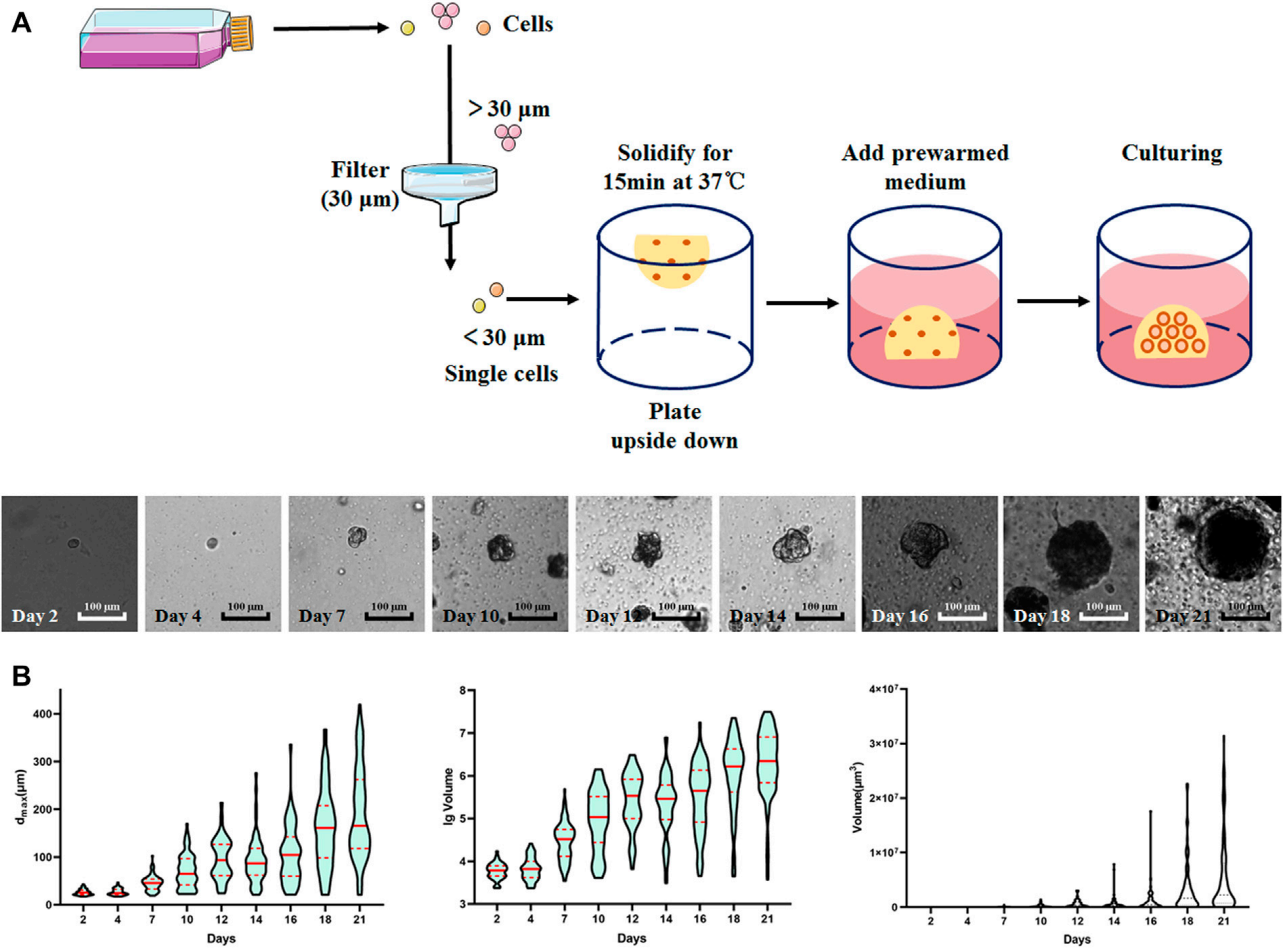
Schematic of spheroids culture procedure and the formation of LNCaP spheroids. **(A)** Schematic of spheroids culture procedure and the images of LNCaP spheroids at different timepoints. **(B)** The violin plots of 3D embedded LNCaP cells/spheroids based on d_max_/volume/lg volume.

A diagram indicating the process for establishing 3D LNCaP spheroids is shown in Figure 1A. The spheroids were initiated from single cells, the formation of LNCaP spheroids was observed from the seventh day, and the spheroid size increased over time (Figure 1A). To measure the continuous changes in the spheroids, we used three parameters (d_max_, volume, and lg volume) to describe the size of the spheroids. Among these three parameters, the d_max_ and lg volumes can better reflect the size change of LNCaP spheroids and were selected to evaluate subsequent drug trials (Figure 1B).

### Drug Interaction Testing of JQ1 Combined With Docetaxel

**FIGURE 2:**
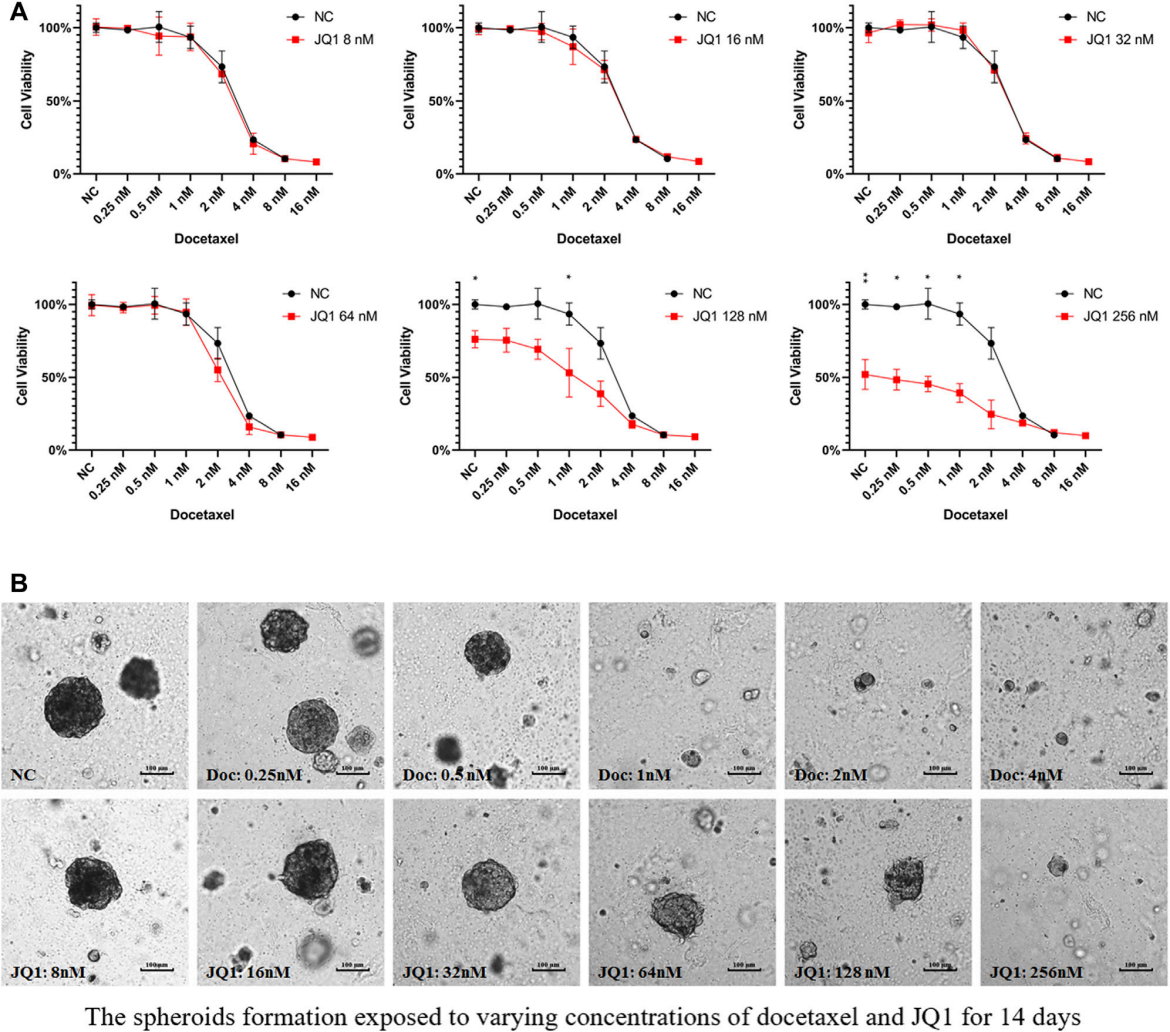
Drug interaction testing of JQ1 combined with docetaxel. **(A)** The cell viability of 2D-cultured LNCaP cells exposed to varying docetaxel concentrations and a fixed concentration of JQ1 (8 nM/16 nM/32 nM/64 nM/128 nM/256 nM). **(B)** The LNCaP spheroids formation exposed to varying docetaxel/JQ1 concentrations for 14 days.

The diagonal method was used to evaluate whether different concentrations of JQ1 could enhance the inhibition of docetaxel. We found that JQ1 significantly enhanced the cell inhibition of docetaxel compared with docetaxel alone at 128 and 256 nM solubility (Figure 2A). We next tested the spheroid formation exposed to varying concentrations of docetaxel and JQ1 for 14 days and found that the formation of LNCaP spheroids could be inhibited when exposed to docetaxel concentrations higher than 1 nM or JQ1 concentrations higher than 128 nM (Figure 2B).

### Cell Viability Analysis of 2D Cells and 3D Spheroids Treated With Drugs

**FIGURE 3:**
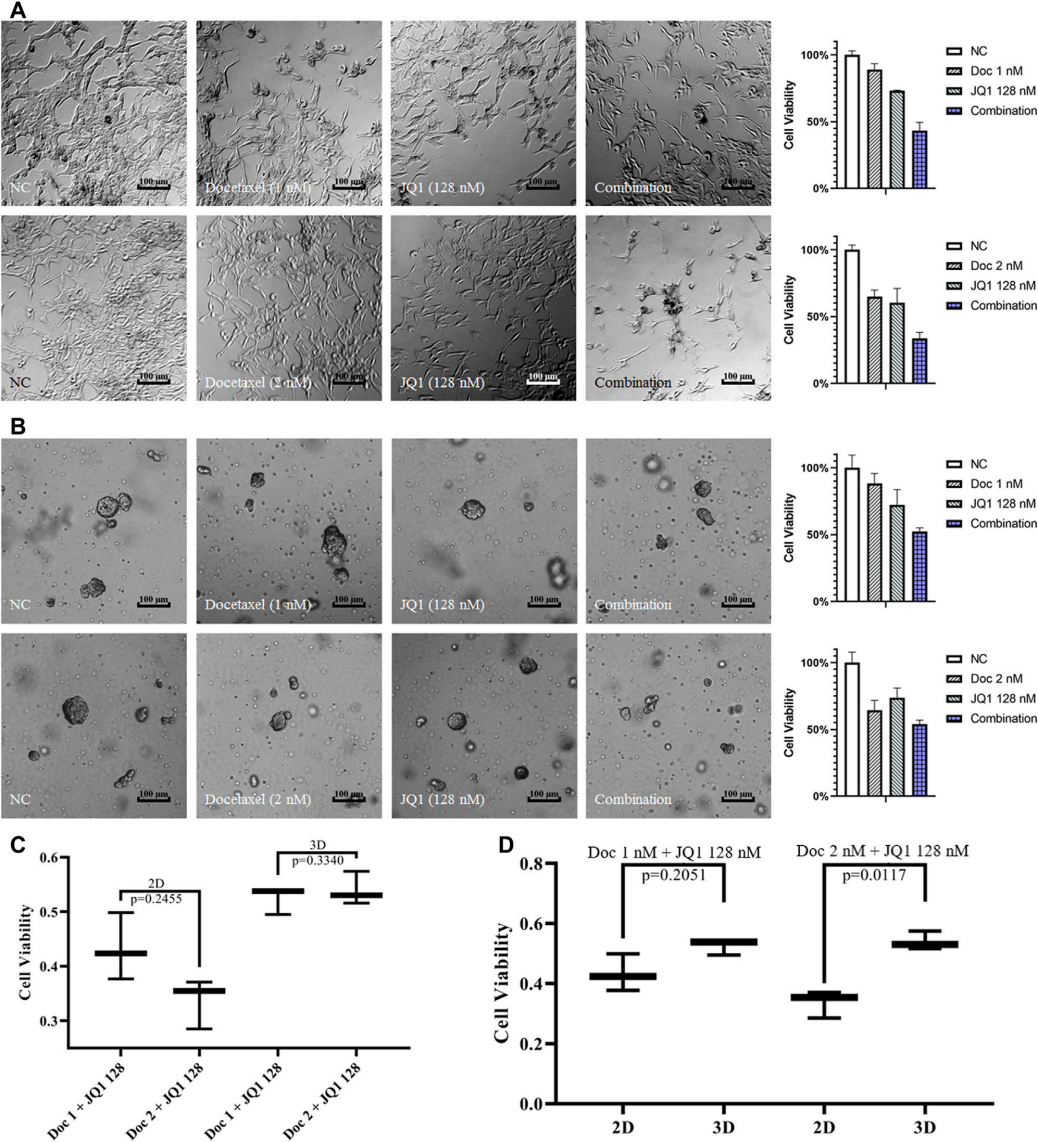
Cell viability analysis of 2D cells and 3D spheroids treated with drugs. **(A)** The images and cell viability of 2D LNCaP cells exposed to 1 nM/2 nM docetaxel and 128 nM JQ1 alone or in combination. **(B)** The images and cell viability of 3D LNCaP cells exposed to 1 nM/2 nM docetaxel and 128 nM JQ1 alone or in combination. **(C)** The cell viability of 2D/3D-embedded cultured LNCaP cells exposed to 1 nM/2 nM docetaxel combined with 128 nM JQ1. **(D)** The cell viability of 2D/3D-embedded cultured LNCaP cells exposed to 128 nM JQ1 combined with 1 nM/2 nM docetaxel.

Based on the initial screening of the above drug solubility, we next analyzed the cell viability of 2D cells and 3D spheroids exposed to JQ1 and docetaxel. In both 2D and 3D cell cultures, the inhibition of cell activity was significantly increased in the JQ1 combined docetaxel group compared to JQ1 or docetaxel treatment alone (Figures 3A,B). Interestingly, compared with JQ1 combined with 1 nM docetaxel, JQ1 combined with 2 nM docetaxel did not significantly increase the inhibition of cell activity, suggesting that JQ1 combined with docetaxel could reduce the solubility of docetaxel (Figure 3C). In addition, the drug resistance of 3D spheroids was significantly higher than that of 2D cells in the 2 nM docetaxel combined with 128 nM JQ1 group (*p* = 0.0117, Figure 3D).

### Formation Characteristic Analysis of 3D LNCaP Spheroids Treated With Drugs

**FIGURE 4:**
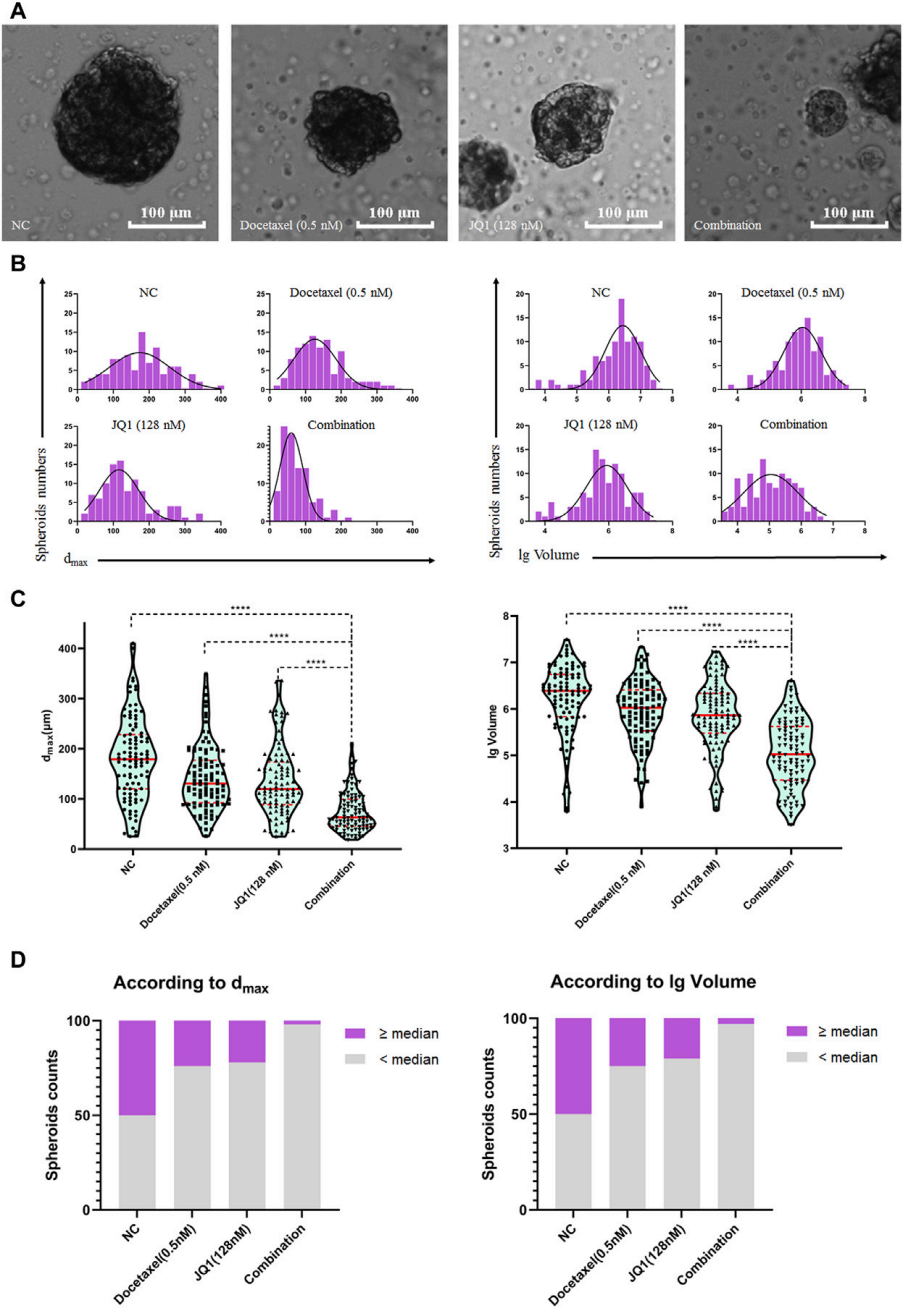
Formation characteristic analysis of 3D LNCaP spheroids treated with drugs. **(A)** The image of the median LNCaP spheroids exposed to 0.5 nM of docetaxel and 128 nM of JQ1 alone or in combination. **(B)** Histogram of d_max_ and the lg volume of LNCaP spheroids exposed to 0.5 nM of docetaxel and 128 nM of JQ1 alone or in combination. **(C)** The violin plot of the LNCaP spheroids distribution (according to d_max_ and the lg volume of LNCaP spheroids). **(D)**. The numbers of LNCaP spheroids bigger than the median spheroids in NC group (according to d_max_ and lg volume).

The 3D spheroid reduces the drug’s contact area compared to 2D cells, but better reflects the physical conditions of the tumor *in vitro*. We then analyzed the formation characteristics of 3D LNCaP spheroids in different drug treatment groups. As shown in Figure 4A, JQ1 with docetaxel inhibited 3D LNCaP spheroid formation compared to JQ1 or docetaxel treatment alone. According to the above test, the d_max_ and lg volume data were further collected from 100 3D LNCaP spheroids of each group, and the frequency distributions and D’Agostino-Pearson omnibus normality tests showed that all the d_max_ and lg volume data were normally distributed (Figure 4B and Table 1).

**TABLE 1:** The results of the normality tests for LNCaP spheroids exposed to docetaxel/JQ1 for 14 days.

Group	d_max_			lg volume		
	D’Agostino-Pearson omnibus (K2)	P-value	Passed normality test (alpha = 0.05)	D’Agostino-Pearson omnibus (K2)	P-value	Passed normality test (alpha = 0.05)
NC	1.761	0.4145	Yes	2.836	0.2422	Yes
Docetaxel	5.019	0.0813	Yes	2.13	0.3447	Yes
JQ1	1.46	0.4819	Yes	0.1604	0.9229	Yes
Combination	0.2779	0.8703	Yes	0.8124	0.6662	Yes

The unpaired tests showed that the median values of d_max_ and lg volume of LNCaP spheroids exposed to the combination treatment were significantly smaller than the d_max_ and lg volume for the single agent treated group and the untreated (NC) group (*p* < 0.0001, respectively, Figure 4C). We also calculated the number of LNCaP spheroids larger than the median value of d_max_ and lg volume in the NC group and found that the number of LNCaP spheroids larger than the NC median exposed to the combination treatment group was significantly less than that in the docetaxel, JQ1, and NC groups (Figure 4D).

## Discussion

Since Prof. Huggins and Hodges first discovered the hormonal dependence of PCa in 1941 (Huggins and Hodges, 1941), hormonal therapy has become the backbone of metastatic prostate cancer treatments. A variety of strategies focusing on blocking androgen-AR signaling are available to treat PCa, and most have been shown to induce significant tumor regression and normalize serum PSA levels (Lochrin et al., 2014). However, almost all patients with mPCa are resistant to hormonal therapy and progress to mCRPC. Deregulated androgen-AR signaling, such as AR amplification, mutation, and altered pathways, can drive CRPC progression (Holzbeierlein et al., 2004). PCa cells may develop resistance after different hormone treatments; thus, new non-AR-dependent treatment strategies should be explored in the future.

BET-inhibitors have rapidly developed in recent years, and some have already entered clinical trials (Alqahtani et al., 2019). BET inhibitors induce cytostatic rather than cytotoxic effects, which indicates that the combination with other drugs might be a better choice in cancer treatment (Pervaiz et al., 2018). JQ1, the groundbreaking BET-inhibitor drug, is a new and effective treatment strategy for patients with mPCa. JQ1 is a potent small-molecule inhibitor of BRD4 that has been shown to reduce the transcription of AR target genes (Seton-Rogers, 2014). It can also reduce the proliferation of PCa cells and organoids with known AR mutations, AR amplification, and AR-V7 expression (Welti et al., 2018). JQ1 is thought to be a potential novel PCa therapy to overcome aberrant AR signaling and improve the outcome of patients beyond current PCa treatments (Welti et al., 2018). However, new literature also showed that JQ1 could promote PCa invasion and metastasis in a BET protein-independent manner when PCa cell growth is inhibited (Wang et al., 2020). Docetaxel is an effective anti-cancer cytotoxic drug agent for patients with mPCa. It has been suggested that the effects of JQ1 could be synergistically amplified by docetaxel addition both *in vitro* and *in vivo* in esophageal adenocarcinoma (Song et al., 2020). On the other hand, this synergistic amplification was not observed when docetaxel was combined with a BRD4-proteolysis targeting chimeric in breast cancer (Noblejas-López et al., 2019).

Our study performed drug testing experiments based on 2D and 3D preclinical models, which showed that the cell growth inhibition by combinational treatment (JQ1 and docetaxel) was significantly higher than that of each treatment alone. The same tendency was also observed for LNCaP spheroid formation. For the first time, we showed that JQ1 and docetaxel are potential combination therapies for patients with PCa.

2D cell culture was introduced as a tool for anti-cancer drug screening in the 1950s (Eagle and Foley, 1958) and has since become an essential part of preclinical drug discovery. 2D-cultured cells are grown as a monolayer, providing a flat “full-on-display” structure, which is different from cells *in vivo*. 2D drug testing experiments showed greater sensitivity than 3D-cultured cells and PDX models. This is one crucial reason why the success rate of novel anti-cancer drugs selected by 2D preclinical models might be so low in clinical trials (Hay et al., 2014; Stock et al., 2016). PDX models and 3D-cultured cells provide more *in vivo*-like preclinical models that better mirror *in vivo* responses (Duval et al., 2017), but the efficiency of PCa PDX/organoid establishment has been relatively low. Spheroids established from suitable PCa cell lines are another effective preclinical model for anticancer drug screening.

Drug-dose-response curves are still widely used to measure anti-cancer drug sensitivity, but they are developed based on drug testing work in 2D monolayer cultured cells. IC50 values were shown to be an imperfect index in 3D drug testing experiments (Berrouet et al., 2020a), indicating that a new evaluation system should be established for spheroids and organoids. In this study, we performed different experiments using 3D embedded-cultured spheroids. We found that d_max_ and lg volume were suitable parameters for LNCaP cells/spheroid size displaying and evaluating cell viability. They should also become suitable indices to assess the efficacy of anti-cancer drug treatment by inhibiting spheroid formation.

This study has some limitations. First, we only investigated the potential combination treatment for PCa. Further projects should systematically explore if this drug combination (JQ1 and docetaxel) is synergistic (such as miniaturized checkerboard assays). In addition, the molecular mechanisms underlying this potential synergistic effect should also be investigated.

## Conclusion

3D cultured spheroid of PCa is an effective tool to study PCa drug trials. JQ1 combined with docetaxel may be an effective treatment for advanced PCa.

## Data Availability

The raw data supporting the conclusion of this article will be made available by the authors, without undue reservation.
